# Myeloid-related protein 8/14 complex describes microcirculatory alterations in patients with type 2 diabetes and nephropathy

**DOI:** 10.1186/1475-2840-8-10

**Published:** 2009-02-20

**Authors:** Klaus Burkhardt, Sonja Schwarz, Chengrui Pan, Felix Stelter, Konstantin Kotliar, Maxilian Von Eynatten, Daniel Sollinger, Ines Lanzl, Uwe Heemann, Marcus Baumann

**Affiliations:** 1 Nephrological Clinic Weissenburg, 91781 Weissenburg, Germany; 2 Department of Nephrology, Technical University Munich, Ismaningerstr. 22, 81675 Munich, Germany; 3 Labor Schottdorf MVZ, 86105 Augsburg, Germany; 4 Department of Ophthalmology, Technical University Munich, Ismaningerstr. 22, 81675 Munich, Germany

## Abstract

**Background:**

Inflammation contributes to cardiovascular complications in type 2 diabetes, which are often characterized by microvascular alterations. We investigated whether myeloid-related protein 8/14 complex (MRP8/14) secreted by transmigrating monocytes and granulocytes may represent a biomarker for microvascular alterations in patients with type 2 diabetes and nephropathy.

**Methods:**

MRP8/14 was measured in 43 patients with type 2 diabetes and nephropathy. Additionally, the inflammatory markers Interleukin-6 (IL-6), Tumor necrosis factor-α (TNF-α) and C-reactive protein (CRP) were quantified. To detect microvascular alterations proteinuria and retinal vessel caliber were used as classical and novel marker, respectively. Proteinuria was quantified by protein-creatinine ratio (PCR); retinal vessel caliber was quantified after retina photography on digitalized retina pictures.

**Results:**

MRP8/14 was positively associated with inflammation (*r *= 0.57), proteinuria (*r *= 0.40) and retinal arterial caliber (*r *= 0.48). Type 2 diabetic patients with MRP8/14 values above the median of 5.8 μg/ml demonstrated higher proteinuria and larger retinal artery caliber than patients with MRP8/14 values below the median (logPCR: -0.51 ± 0.52 versus -0.96 ± 0.46, *P *< 0.01; retinal artery lumen (μm): 178.3 ± 14.1 versus 162.7 ± 14.9 *P *< 0.01). Both groups did not differ with regard to metabolic factors and blood pressure. MRP8/14 was an independent predictor of retinal artery caliber in multivariate stepwise regression analysis (*β *= 0.607) and was positively associated with IL-6 (*r *= 0.57, *P *< 0.001) and TNF-α (*r *= 0.36, *P *< 0.05).

**Conclusion:**

MRP8/14 – a marker for transendothelial migration – describes not only the state of inflammation in diabetic nephropathy, but additionally the degree of microvascular alterations in the glomerular and retinal bed. Therefore, MRP8/14 may be a potentially selective novel biomarker for microcirculatory defects in diabetic nephropathy.

## Introduction

Chronic inflammation contributes to type 1 and type 2 diabetes mellitus and is involved in the development of cardiovascular complications [1,2]. Microvascular complications, which are largely defined by the presence and degree of proteinuria, are associated with inflammation [3]. In the case of diabetic nephropathy proteinuria and systemic inflammation define the cardio-vascular risk [4]. It would be interesting to correlate these changes to the microvasculature.

Retinal vessels represent a direct non-invasive measurement of vascular lumen diameter on the level of resistance arteries, hence the microcirculatory level [5]. In this respect an enlarged retinal arteriolar and venular caliber is related to diabetes and systemic inflammation [6,7]. The dilation of the retinal vessels has been attributed to an increased production of nitric oxide as a result of up-regulated nitric oxide synthase messenger RNA, secondary to release of cytokines such as IL-1 and TNF-α [8].

Apart from these direct effects of inflammatory markers on the microcirculation in diabetic nephropathy, little is known about the contribution of inflammation-initiated transmigration of monocytes through the arteriolar wall. We hypothesize that the myeloid-related protein 8/14 complex (MRP8/14), a marker of monocyte and neutrophil activation [9], predicts microvascular changes at an early stage. MRP8/14, also termed calprotectin, leukocyte protein L1 complex or cystic fibrosis antigen, is a heterodimer of two calcium-binding proteins (S100A8 and S100A9, also referred to as MRP8 and MRP14, or calcranulin A and B involved in calcium-dependent signaling, cell differentiation, cell cycle progression, and cytoskeleton-membrane interactions [10]. MRP8 and MRP14 are primarily expressed in cells of myeloid origin, particularly in monocytes and neutrophils [11]. Upon phagocyte activation, MRP8 and MRP14 form the MRP8/14 complex, which translocates to the cytoskeleton and plasma membrane, where it is secreted [12,13]. This is an early event during transendothelial migration and thus represents an interaction of MRP-expressing neutrophils and monocytes with endothelium [14]. Therefore we further hypothesize that systemic inflammation in diabetic nephropathy results in an increased transendothelial migratory activity of monocytes and neutrophils and hence microvascular changes in the glomerular and retinal vascular bed. It is the aim of this study to investigate in patients with type 2 diabetes and nephropathy whether the degree of proteinuria and retinal arteriolar lumen diameter are associated with markers of inflammation and endothelial transmigration. Furthermore we describe one patient demonstrating reduction of MRP8/14 and proteinuria under TNF-blockade, potentially mirroring the pathophysiological relevance of MRP8/14.

## Methods

Forty-three patients with type 2 diabetes with nephropathy were recruited from the nephrological clinic Weissenburg. In the entire cohort, the simplified MDRD (Modification of Diet in Renal Disease) equation was used to estimate the glomerular filtration rate (eGFR (ml/min/1.73 m^2^) = 186 × (serum creatinine (mg/dl))^-1.154 ^× (age)^-0.203 ^× (0.742 if female) × (1.212 if black)). Proteinuria was quantified in spot urine by measurement of the protein-creatinine ratio (PCR). PCR was log-transformed to better approximate normal distributions. Additionally, cardiometabolic risk factors were evaluated and biological parameters measured, according to the recommendations of the American and European clinical practice guidelines. Anti-diabetic and anti-hypertensive medication was recorded including insulin-therapy, oral anti-diabetics, and the total number of antihypertensive drugs including differentiation of inhibitors of the renin-angiotensin system (RAS) or calcium channel blockers. The research has been carried out in accordance with the Declaration of Helsinki (2000) of the World Medical Association, and has been approved by the local Ethics Committee of the institution. Written informed consent was obtained from each patient after full explanation of the purpose, nature and risk of all procedures. One trained person measured anthropometric characteristics. For ≥3 hours before the examination, the participants refrained from heavy exercise, smoking, and alcohol or caffeine-containing beverages. The body mass index was weight in kilograms divided by height in meters squared. Venous blood samples, collected after overnight fasting, were analyzed by standard automated methods for lipids and blood glucose. Diabetes mellitus was defined as a fasting blood glucose level of at least 7.0 mmol/l or as the use of antidiabetic drugs.

IL-6, TNF-α (R&D Systems, Minneapolis, MN, USA) and MRP8/14 (Bühlmann Laboratories, Schönenbuch, Switzerland), were determined by commercially available ELISA using serum samples.

### Measurement of retinal vessels

One trained person performed the retina measurements. Methods used to measure and summarize retinal vessel diameters from digitized photographs followed a standardized protocol described elsewhere [15]. In brief, two 30° color retinal photographs of the left and two photographs of the right eye were taken at baseline. They were digitized by a high resolution scanner with standard settings (IMEDOS, Jena, Germany). Graders masked to the characteristics of participants used a computer program to measure diameters of all arterioles and venules in a specified zone surrounding the optic disc. These measurements were combined into summary indices – the central retinal arteriolar and venular equivalents – which represented the average arteriolar (CRAE) and venular (CRVE) diameters of that eye, respectively. These were additionally expressed as the retinal arteriolar-venular ratio (AVR). The ratio compensated for possible magnification differences between eyes, and an AVR of 1 indicated that arteriolar diameters were, on average, the same as venular diameters in that eye, while a smaller ratio suggested narrower arterioles. The AVR reflects the mean of the four photographs.

### Aortic Pulse Wave Velocity

Aortic pulse wave velocity (aPWV) was assessed by sensitive transducers and the results were analysed using the Complior program (Complior, Artech Medical, Pantin, France) [16]. First, patients rested in the supine position for 5 min. Brachial blood pressure was measured by an oscillometric device (Omron, Japan). Pressure-sensitive sensors were placed on the right carotid, radius and femoral artery. The distance between two sites (carotid-radius, carotid-femoral) was estimated automatically according to the body height of the patients. The time difference was determined from the delay of starting phase of the first wave between carotid and another site ("foot-to-foot method"). Aortic PWV was calculated by dividing the distance by the time difference. At least 10 signals were obtained twice and averaged to the results. In the case of an intra-patient variance of more than 10%, a third measurement was performed. Data were collected by a single observer (P.C.R.). The intra-observer coefficient of variation for carotid-femoral (C-F) PWV was 2.5%.

### 24-hour ambulatory blood pressure measurements

Programmed and validated blood pressure devices (model 90207; Spacelabs Medical, Issaquah, Washington, USA) were used to obtain blood pressure recordings at intervals of 20 min from 0700 to 2300 h, and every 30 min from 2300 to 0700 h. We calculated the within-subject 24-h means of the ambulatory measurements with weights according to the time interval between successive readings [17].

### Statistical analysis

Data are expressed as means ± SD or percentages. The unpaired t-test, Mann-Whitney U-test or χ^2^-test were used for comparing the upper and lower MRP8/14 median group. Within the cohort, multiple linear regression analysis was used to evaluate which factors were independently associated with retinal arteriolar diameter. Data were analyzed using SPSS software (version 14.0). All tests were two-sided and a *P*-value < 0.05 was considered significant.

## Results

### Baseline characteristics

Patients with diabetic nephropathy and proteinuria are characterized in Table 1. They were stratified according to the median of the myeloid-related protein complex to assess whether MRP8/14 affects cardiovascular and metabolic characteristics (MRP8/14: median: 5.8 μg/ml, 25^th ^percentile: 3.2, 75^th ^percentile: 11.8, Table 1).

**Table 1:** Clinical characteristics of patients with type 2 diabetes and nephropathy as total cohort and grouped according to the median of MRP8/14

**Characteristics**	**ALL (*n *= 43)**	**< MRP8/14 median (*n *= 22)**	**> MRP8/14 median (*n *= 21)**
Age (years)	66.5 ± 12.2	65.2 ± 13.6	67.3 ± 12.0
Gender (% men)	58.1	68.2	47.6
BMI (kg/m^2^)	31.2 ± 5.9	29.8 ± 3.9	32.3 ± 7.6
Waist circumference (cm)	110.7 ± 15.3	107.1 ± 10.4	114.4 ± 19.1
Smoking (%)	6.9	13.6	0.0
Glycemia (mmol/l)	6.55 ± 1.95	6.64 ± 1.46	6.43 ± 1.86
Hba1c (%)	6.7 ± 0.8	6.7 ± 0.8	6.7 ± 0.7
Cholesterol (mmol/l)			
Total	180.2 ± 37.6	171.2 ± 33.6	185.1 ± 38.5
HDL	49.1 ± 13.6	46.4 ± 9.8	51.2 ± 13.2
LDL	100.2 ± 31.6	96.9 ± 28.9	104.1 ± 35.6
Triglyceridemia (mmol/l)	174.9 ± 113.2	175.6 ± 94.8	146.1 ± 88.7
eGFR (ml/min/1.73 m^2^)	61.3 ± 36.1	66.5 ± 30.0	55.1 ± 44.8
Protein-creatinine ratio (log)	-0.76 ± 0.54	-0.96 ± 0.46	-0.51 ± 0.52 **
			
Insulin therapy (%)	32	17	50 *
RAS-Inhibition (%)	86	91	85
Statin therapy (%)	45	57	35
			
Cardiovascular			
24 h systolic BP (mmHg)	135.6 ± 11.5	134.1 ± 11.6	138.3 ± 11.4
24 h diastolic BP (mmHg)	71.8 ± 10.9	72.6 ± 11.8	68.4 ± 10.0
24 h Pulse pressure (mmHg)	63.8 ± 8.4	62.5 ± 7.7	66.5 ± 8.5
aPWV	11.5 ± 3.1	11.7 ± 3.3	11.4 ± 2.9
CRAE (μm)	169.1 ± 16.0	162.7 ± 14.9	178.3 ± 14.1 **
CRVE (μm)	219.8 ± 22.3	217.4 ± 26.7	221.3 ± 19.2
AVR	0.818 ± 0.085	0.796 ± 0.077	0.856 ± 0.082 *
			
Inflammation and transmigration			
IL-6 (pg/ml)	1.01 ± 1.09	0.59 ± 0.31	1.70 ± 1.52 *
TNF-α (pg/ml)	6.95 ± 4.52	5.05 ± 3.62	9.10 ± 5.27 *
CRP (mg/dl)	9.5 ± 13.2	5.3 ± 6.5	15.5 ± 19.1
MRP8/14 (μg/ml)	8.53 ± 6.92	3.98 ± 1.04	14.08 ± 7.73 ***

Values represent mean ± SD or percentages (%).

BMI: body mass index, eGFR: estimated glomerular filtration rate, HDL: High-density lipoprotein, LDL: Low-density lipoprotein, IL-6: Interleukin-6, TNF-α: Tumor necrosis factor-α, MRP8/14: myeloid-related protein complex, CRP: C-reactive peptide, BP: blood pressure, RAS: renin-angiotensin system, AVR: arteriolar-venular ratio, CRAE: central retinal arteriolar equivalent, CRVE: central retinal venular equivalent *p < 0.05, **p < 0.01 ***p < 0.001 versus < MRP8/14 median

Anthropometric data and renal function were comparable between the groups. The distribution according to the median of MRP8/14 did not lead to difference in HbA1c and lipid profile. The use of insulin therapy was significantly higher in patients with higher MRP8/14 values (50 versus 17%, *P *< 0.05). The number of antihypertensive drugs and the use of calcium channel blocker (data not shown) and RAS-blocker were similar in both groups. MRP8/14 values had no influence on ambulatory blood pressure and arterial stiffness. In contrast, MRP8/14 values above the median correlated with microvascular alterations including increased proteinuria and enlarged retinal arterioles. Proinflammatory characteristics were prominent in patients with MRP8/14 values above the median.

### Association of inflammation and microcirculatory aspects

**Figure 1:**
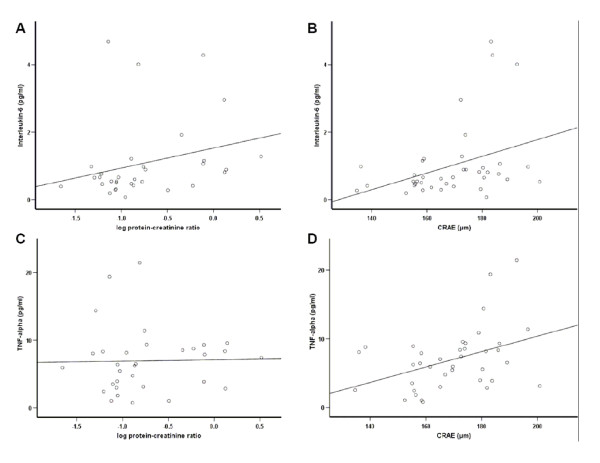
**Bivariate correlations of Interleukin-6 (IL-6) and TNF-α with proteinuria and retinal artery caliber in patients with type 2 diabetes and nephropathy: A) IL-6 and proteinuria, B) IL-6 and retinal artery caliber, C) TNF-α and proteinuria and D) TNF-α and retinal artery caliber**. We observed significant positive correlations for A) r = 0.39, B) r = 0.42 and D) r = 0.41. All correlations are given as calculated by Spearman correlation coefficient.

Patients with diabetic nephropathy and proteinuria had a positive association between the degree of proteinuria and inflammation, although restricted to IL-6 (*r *= 0.39, *P *< 0.05, Figure 1). Other inflammatory parameters such as TNF-α and CRP were not significantly associated with the degree of proteinuria.

As second microvascular characteristic the luminal diameters of central retinal arterioles (CRAE) were investigated and associated with inflammatory markers. Here, IL-6 and TNF-α were positively associated with the lumen diameter of CRAE (*r *= 0.42, *P *< 0.01 and *r *= 0.41, *P *< 0.05).

### Association of transmigration, inflammation and microcirculatory aspects

**Figure 2:**
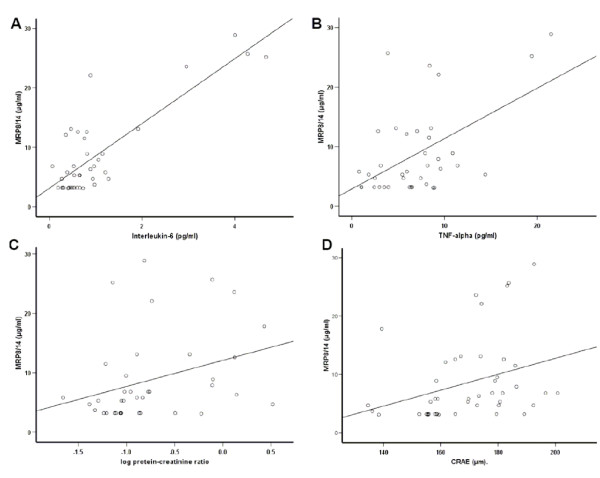
**Bivariate correlations of MRP8/14 with A) Interleukin-6 (IL-6) and B) TNF-α as inflammatory marker and C) with proteinuria and D) retinal artery caliber as microcirculatory marker in patients with type 2 diabetes and nephropathy**. We observed significant positive correlations for A) r = 0.57, B) r = 0.36, C) r = 0.40 and D) r = 0.48. All correlations are given as calculated by Spearman correlation coefficient.

MRP8/14 as marker for monocyte transmigration was positively associated with IL-6 and TNF-α (*r *= 0.57, *P *< 0.001 and *r *= 0.36, *P *< 0.05, Figure 2). Moreover, MRP8/14 was positively associated with the degree of proteinuria (*r *= 0.40, *P *< 0.05) and the lumen diameters of CRAE (*r *= 0.48, *P *< 0.01).

Multivariable regression models were used to estimate the partial association between CRAE, inflammatory markers and MRP8/14 (Table 2). In patients with diabetic nephropathy and proteinuria, IL-6, TNF-α and MRP8/14 were significantly and independently associated with CRAE after adjustment for age, gender, BMI, glucose, 24 h SBP, and eGFR. This association remained significant for MRP8/14 and CRAE even after further adjustment for IL-6 and TNF-α.

**Table 2:** Multivariate stepwise regression analysis for the central retinal artery equivalent (CRAE).

**Model**	**Coefficient**	**SE**	**β**	***P***
**Model + TNF-α: adjusted R^2 ^= 0.17, P < 0.05^a^**				
Constant	157.628	5.306		< 0.001
TNF-α	1.387	0.617	0.458	< 0.05
**Model + IL-6: adjusted R^2 ^= 0.34, P < 0.01^a^**				
Constant	156.235	4.357		< 0.001
IL-6	5.839	2.296	0.494	< 0.05
**Model + MRP8/14: adjusted R^2 ^= 0.251, P < 0.01^a^**				
Constant	158.702	4.327		< 0.001
MRP8/14	1.061	0.357	0.535	< 0.01
**Model + IL-6 + TNF-α + MRP8/14: adjusted R^2 ^= 0.251, P < 0.01^a^**				
Constant	158.702	4.327		< 0.001
MRP8/14	1.139	0.343	0.607	< 0.01

aModel adjusted for age, gender, BMI, glucose, 24 h SBP, eGFR.

### Effect of TNF-blockade on MRP8/14 and proteinuria

A 48 year-old male patient with type 2 diabetes and nephropathy a HLA-B27 positive ankylosing spondylarthritis was diagnosed. Based on the activity of the ankylosing spondylarthritis a TNF-blockade with etanercept (50 mg/week s.c.) was initiated. Under this treatment we observed after 3 month of treatment that MRP8/14 as transmigratory marker decreased from 19.9 μg/ml prior etanercept to 3.2 μg/ml. After one year of treatment MRP8/14 remained low with 2.2 μg/ml. Secondly, we observed that proteinuria decreased under the TNF-blockade from 378 to 58 mg protein/g creatinine at 3 month and remained low after one year of treatment (49 mg protein/g creatinine).

## Discussion

Our study demonstrates in patients with type 2 diabetes and nephropathy that MRP8/14, a marker for transendothelial migration of monocytes and neutrophils, may predict changes in the microvascular bed of glomeruli and retina beyond inflammation. These changes are for the glomerular bed the degree of proteinuria and in the retinal bed an increase of arteriolar lumen caliber. Thus, MRP8/14 is a potential biomarker for microvascular defects in patients with diabetic nephropathy which may represent a pathophysiological relevant feature. Furthermore, these results implicate a potential deleterious role of increased arteriolar lumen caliber if associated with inflammation and transmigration in patients with diabetic nephropathy.

Diabetic nephropathy is characterized by proteinuria and low-grade systemic inflammation [18]. Both factors have been associated with microvascular complications [1] and hence predict cardiovascular outcome [2]. In this context, MRP8/14 was in our study positively associated with Interleukin-6 as marker for systemic inflammation [19] and TNF-α as marker for metabolic driven inflammation [20] in patients with diabetic nephropathy. This is of relevance as sequential migration of neutrophils is stimulated by TNF-α induced inflammation and can be reduced by inhibition of Interleukin-6 secretion [21]. Thus, a causal relationship with the option to modulate the degree of microvascular damage may be suggested. Besides, the degree of proteinuria and retinal arteriolar caliber as conventional and novel microvascular factors were positively associated with MRP8/14. Thus, systemic MRP8/14 values seem to reflect not only the grade of inflammation, but also may define the microcirculation as the place of action. The microcirculatory action is not restricted to one organ as glomerular and retinal effects are observed. Moreover, Alwegg et al. demonstrated the local coronary release of MRP8/14 in acute coronary syndrome [14]. Thus we suggest that the MRP8/14 activity reflects a generalized microcirculatory effect of increased transendothelial migration. In contrast, there is a lack of association between arterial stiffness measured by the gold standard of aortic PWV and MRP8/14. This suggests that no dominant effect of transmigration is present in the context of the macrocirculation. This further emphasizes MRP8/14 as a novel and potentially selective marker for microcirculatory effects in diabetic nephropathy.

Studies in diabetic nephropathy are fraught with difficulty, given the recognized associations with hypertension and dyslipidemia, both of which are known to influence the microcirculation [22]. It is important to notice that our cohort after stratification according to MRP8/14 remains comparable for the lipid profile and 24-hour blood pressure measurement. Therefore, it is unlikely that MRP8/14 as marker for transendothelial migration is largely influenced by these two factors. Hence, enhanced MRP8/14 may describe a pathomechanism independent of hypertension and dyslipidemia in patients with diabetic nephropathy.

Furthermore, we describe one patient in whom TNF-blockade was initiated due to an ankylosing spondylarthritis. In this patient MRP8/14 and proteinuria measurements were available before and during treatment covering a period of 18 month. The measurements showed a decrease both of transmigration and proteinuria as marker for microcirculatory damage. These observations may be interpreted as pathophysiological relevant link between inflammation, transmigration and microcirculatory alterations. However, in the future this clearly needs to be investigated in detail.

Is a larger retinal arteriolar caliber in patients with diabetic nephropathy deleterious? In patients with type 2 diabetes the myogenic responsiveness is impaired. Schofield et al. demonstrated in pressure myograph experiments using resistance arteries of type 2 diabetic subjects that above a luminal pressure of 50 mmHg no reactive contractility was present whereas control subjects gave a blood pressure dependent myogenic response [22]. As a consequence wall stress may rise resulting in vascular hypertrophy [23].

In our cohort of patients with diabetic nephropathy high MRP8/14 values are associated with larger arteriolar lumen caliber, which may reflect a failure to autoregulate blood flow efficiently [24]. This may increase high blood pressure flow to target organs, effecting downstream damage, as determined in our patients by increased proteinuria particularly in subjects with elevated MRP8/14 levels. The failure of blood flow autoregulation may result in enlarged preglomerular afferent arterioles. This increases intraglomerular pressure with the consequence of proteinuria [25]. Secondly, capillary transmigration may directly damage podocytes thereby aggregating capillary leakage which corresponds with proteinuria [26]. These options raise the new speculation whether the defective myogenic response observed in type 2 diabetes is not only regulated by metabolic factors [27,28], but is also related to the degree of local inflammation and transmigration.

In summary, we demonstrate that MRP8/14 as marker for transendothelial migration describes not only the state of inflammation in diabetic nephropathy, but additionally the degree of microvascular alterations. Therefore, MRP8/14 is a potentially selective novel biomarker for microcirculatory defects, which may play a pathophysiological relevant role.

## Competing interests

The authors declare that they have no competing interests.

## Authors' contributions

KB designed, coordinated and wrote the manuscript. AB carried out the molecular genetic studies. SS performed retinal measurements. CRP performed macrocirculatory measurements.

FS carried out the MRP measurements. KK analyzed the retina measurements. ME performed statistical analysis. DS performed ELISAs. IL analyzed the retina measurements. UW designed, coordinated the manuscript. MB designed, coordinated and wrote the manuscript. All authors read and approved the final manuscript.
